# 

**Published:** 1901-11-15

**Authors:** 


### ANNOUNCEMENTS.

### OHIO STATE BOARD OF DENTAL EXAMINERS.

The next meeting of this Board will be held in the Great Southern Hotel, Columbus, Ohio, beginning Tuesday November, 26th, at 2 p.m. All candidates must bring excavators, burs and filling materials.

For examination blank address the secretary, L. P. Bethel, Kent, Ohio.

---

### PROGRAM FOR THE OHIO STATE DENTAL SOCIETY.

President's Address, H. F. Harvey; Diagnosis in Dental Practice, J. Taft; Nitrous Oxid and Oxygen, H. Ziegler; Technic and Taste, H. B. Mitchell; Cohesion of Gold, C. A. Hawley; Why You Failed with Cataphoresis, W. A. Price; Will a Higher Preliminary Requirement Remove Existing Evils? J. S. Junkerman; Haemophillia, S. D. Ruggles.

THE FOLLOWING CLINICS WILL BE PRESENTED.

Strengthening Porcelain Crowns, C. A. Hawley; Nitrous Oxid in Dental Therapeutics, W. I. Jones; Details in Crown Construction, L. E. Custer; Porcelain Crown and Bridge Work, H. E. Jenkins; Tipping Tooth with Solid Gold Cusp, E. M. Cook; A Method of Constructing Removable Bridges, E. C. Beggs; Combination of Soft and Cohesive Gold in Filling, A. F. Miller; Cavity Preparation for Amalgam, A. F. Miller; Modeling and Swedging Cusps, E. B. Lodge; Logan Crowns, A. S. Condit; Gold Filling in Porcelain Teeth, Otto Arnold; An Emergency Crown, J. W. Harris; Exhibit of New Things, C. H. Burkett; Cap and Logan Crown, S. M. Weaver; Management of Regulating Appliances, V. E. Barnes; Gold Filling, W. T. Born; Pulp Capping, W. T. McLean; Porcelain Inlays, D. W. Clancy; Adapting Platinum Bands and Posts to Porcelain Crowns without Solder, D. Stearn; Porcelain Work, W. O. Hulick; Microscopic Exhibit, J. R. Callahan, W. H. Van Deman, J. F. Stephan, L. P. Bethel; Selected Clinic, W. A. Price.

### INSTITUTE OF DENTAL PEDAGOGICS.

The ninth annual meeting of the Institute of Dental Pedagogics will convene on Tuesday, December 31, 1901, and continue for three days, at the Seventh Avenue Hotel, Pittsburg, Pa. The usual New Year Day railroad rates can generally be obtained. A partial program is submitted:

President's address, by G. E. Hunt, Indianapolis; "Conduct of Operatory Clinic" (a method of keeping records, grades, etc.), by G. V. Black, Chicago; "Executive Work of the Faculty" (a symposium), by Drs. Kirk, Patterson, Stubblefield and Hart; "Metallurgy: How to Teach It," by Dr. Hodgen, San Francisco; "Class-room Methods of Teaching" (a symposium), by Drs. Hoff, Nones, Tenney and Foster; "Teaching Prosthetic Dentistry," by G. H. Wilson, Cleveland; "Bacteriology: How to Teach It," by W. R. Blue, Louisville. Report of committee on Operative and Prosthetic Technics.

D. M. Cattell,
*Chairman Ex. Board.*

